# Guidance Level for Brevetoxins in French Shellfish

**DOI:** 10.3390/md19090520

**Published:** 2021-09-15

**Authors:** Nathalie Arnich, Eric Abadie, Zouher Amzil, Marie-Yasmine Dechraoui Bottein, Katia Comte, Estelle Chaix, Nicolas Delcourt, Vincent Hort, César Mattei, Jordi Molgó, Raphaele Le Garrec

**Affiliations:** 1 Risk Assessment Directorate, ANSES (French Agency for Food, Environmental and Occupational Health and Safety), 94701 Maisons-Alfort, France; estelle.chaix@anses.fr; 2 MARBEC (MARine Biodiversity, Exploitation and Conservation), Université de Montpellier, CNRS, Ifremer, IRD, 34200 Sète, France; eric.abadie@ifremer.fr; 3 Ifremer (French Research Institute for Exploitation of the Sea), 44311 Nantes, France; zouher.amzil@ifremer.fr; 4 Université Côte d’Azur, CNRS, ECOSEAS, UMR7035, Parc Valrose, 06108 Nice, France; y.bottein@gmail.com; 5 UMR7245 MCAM CNRS-MNHN, Muséum National d’Histoire Naturelle, 75231 Paris, France; kcomte@mnhn.fr; 6 Poison Control Centre, Toulouse-Purpan University Hospital and Toulouse NeuroImaging Centre (ToNIC), INSERM 1214, 31059 Toulouse, France; delcourt.n@chu-toulouse.fr; 7 Laboratory for Food Safety, Pesticides and Marine Biotoxins Unit, ANSES (French Agency for Food, Environmental and Occupational Health and Safety), 94701 Maisons-Alfort, France; vincent.hort@anses.fr; 8 University of Angers, INSERM, CNRS, MITOVASC, Equipe CarMe, SFR ICAT, 49100 Angers, France; cesar.mattei@univ-angers.fr; 9 CEA (French Alternative Energies and Atomic Energy Commission), INRAE, University of Paris-Saclay, DMTS, SIMoS, ERL CNRS 9004, 91191 Gif sur Yvette, France; jordi.molgo@cea.fr; 10 Laboratoire Interactions Epitheliums Neurones (LIEN), University of Brest, 29200 Brest, France; raphaele.legarrec@univ-brest.fr

**Keywords:** brevetoxins, neurotoxic shellfish poisoning, shellfish, guidance level, marine biotoxins, emerging toxins

## Abstract

Brevetoxins (BTXs) are marine biotoxins responsible for neurotoxic shellfish poisoning (NSP) after ingestion of contaminated shellfish. NSP is characterized by neurological, gastrointestinal and/or cardiovascular symptoms. The main known producer of BTXs is the dinoflagellate *Karenia brevis*, but other microalgae are also suspected to synthesize BTX-like compounds. BTXs are currently not regulated in France and in Europe. In November 2018, they have been detected for the first time in France in mussels from a lagoon in the Corsica Island (Mediterranean Sea), as part of the network for monitoring the emergence of marine biotoxins in shellfish. To prevent health risks associated with the consumption of shellfish contaminated with BTXs in France, a working group was set up by the French Agency for Food, Environmental and Occupational Health & Safety (Anses). One of the aims of this working group was to propose a guidance level for the presence of BTXs in shellfish. Toxicological data were too limited to derive an acute oral reference dose (ARfD). Based on human case reports, we identified two lowest-observed-adverse-effect levels (LOAELs). A guidance level of 180 µg BTX-3 eq./kg shellfish meat is proposed, considering a protective default portion size of 400 g shellfish meat.

## 1. Introduction

Brevetoxins (BTXs) are a group of lipophilic marine biotoxins mainly produced by the dinoflagellate *Karenia brevis*, which forms harmful algal blooms known as red tides. BTXs can accumulate in shellfish, fish and other marine organisms, and be aerosolized from marine waters [1,2,3]. More than 70 analogues and metabolites of BTXs have been reported, most of them are issued from their metabolism in shellfish [1]. BTX-2 is the major analogue produced by *K. brevis*, while BTX-3 was reported at higher level than BTX-2 in shellfish, i.e., in oysters (*Crassostea gigas*) and cockles (*Austrovenus Stutchburyi*) in New Zealand [4,5,6]; in horse conch (*Triplofusus giganteus*), lightning whelk (*Sinistrofulgur sinistrum*), banded tulip (*Cinctura hunteria*), fighting conch (*Strombus alatus*), pear whelk (*Fulguropsis spirata*), clam (*Mercenaria* spp.) and oyster (*Crassostrea virginica*) in Florida [7,8,9]. Humans can be exposed to BTXs through seafood consumption, inhalation, and cutaneous contact. The symptoms associated with BTX intoxication and their duration depend in part on these routes of exposure. According to a recent systematic review of the literature carried out by Young et al. 2020 [10], BTXs account for 7.9% of the studies (30/380 publications from 1985 to 2019) on human health effects associated with the efflorescence of marine microalgae and their toxins.

Among the health effects of BTXs in humans, the syndrome called “Neurotoxic Shellfish Poisoning” (NSP) refers to poisoning associated with the ingestion of molluscs contaminated with BTXs [3]. A few hundred cases of NSP have been described so far in peer-reviewed articles. The small number of such events may be due to the introduction of regulations on the marketing of shellfish, based on the monitoring of *K. brevis* cells in marine water, and BTXs in shellfish along the impacted coasts of the USA, Mexico, Australia and New Zealand [11,12,13,14]. Nonetheless, the number of NSP cases remains underestimated, including in Florida where the reporting of this poisoning is mandatory. This underestimation may be due to misdiagnosis and under-reporting. In addition, warning communications seem to be insufficient concerning the practice of recreational harvesting. Indeed, a retrospective study conducted in Florida between 2004 and 2009 revealed that 78% of NSP cases occurred in non-residents (tourists), and residents of Florida living away from the coast [15]. The United States, Australia, New Zealand, and Mexico apply a threshold of 800 μg BTX-2 equivalents/kg shellfish flesh [11,12,13,14]. In the Codex Alimentarius standard for live and raw bivalve molluscs (CODEXSTAN 292–2008, rev. 2015) [16], the maximum level for BTXs is 200 mouse units (MU) or equivalent per kg of mollusc flesh. A MU is the amount of raw extract required to kill 50% of mice using a mouse bioassay [17]).

**Figure 1:**
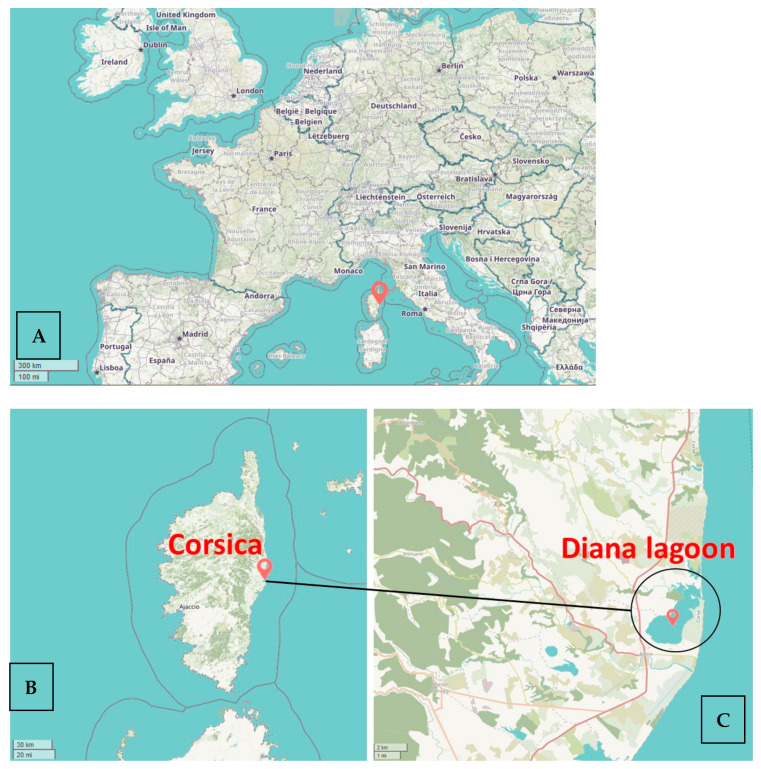
Location of the Diana lagoon in Corsica Island, Mediterranean Sea (red bookmark, © OpenStreetMap contributors. Tile style for the Humanitarian OpenStreetMap Team hosted by OpenStreetMap France). (**A**) Location of Corsica Island in Europe. (**B**) Location of Diana lagoon in Corsica Island. (**C**) Enlargement of the area of the Diana lagoon.

Although these marine biotoxins pose a proven health risk in some regions of the world such as in Florida, they constitute an emerging risk in France and more broadly in Europe, where they are not regulated. It was thanks to the French network for monitoring the emergence of marine biotoxins in shellfish (EMERGTOX) that BTXs were first detected in November 2018 in French mussels from a lagoon (Diana lagoon) in the Corsica Island, Mediterranean Sea (Figure 1) [18], and subsequently regularly every year during the cold seasons (autumn, winter). The maximum reported concentration was 345 µg/kg in the mussel digestive gland for the sum of BTX-2 and BTX-3 in November 2020 (corresponding to an estimated value of 57 µg/kg of total flesh). In addition, water samples obtained from the same site to look for microalgae that are potential BTX producers identified *Karenia mikimotoi*, *Karenia papilionacea*, *Karenia longicanalis* and an undescribed species *Karenia* sp. 1. Other potential BTX producers were found among the raphidophytes, and the species identified were *Fibrocapsa japonica* and *Heterosigma akashiwo* [18]. More research is needed to identify the one or more taxa responsible for the contamination of mussels with BTXs in Corsica.

BTXs are neurotoxins that primarily target the voltage-gated Na^+^ channels (Na_V_) [19,20,21]. Na_V_ channels are a class of transmembrane proteins that open their pore in response to membrane depolarization, allowing the influx of Na^+^ that initiates the generation of action potentials in excitable cells [22]. The binding of BTXs to the site-5 [23] of the Na_V_ channel α subunit produces persistent channel activation by shifting the activation to more negative potentials, and slows the inactivation process of these channels [24,25]. Therefore, BTXs can be considered Na_V_ channel activators, like ciguatoxins [26], a group of marine biotoxins responsible of ciguatera poisoning. BTXs depolarize neuronal and muscle membranes, and consequently promote their excitability, as well as Ca^2+^-dependent mechanisms. A variety of mammalian Na_V_ channel isoforms have been identified, and are expressed in distinct tissues [27,28]. The specific affinity of BTXs for Na_V_ channel subtypes and the tissue distribution of Nav channels [28,29,30] can explain the primarily neurological nature of the symptoms observed in humans and animals, which involve the central and peripheral nervous, but also gastrointestinal and cardiovascular systems. There is no specific antidote for NSP; the treatment is therefore symptomatic.

Three main types of methods can be used to analyze BTXs in microalgae cells, seawater samples, marine organisms (molluscs, fish), and sea spray/aerosol samples. (1) Physico-chemical methods such as liquid chromatography-mass spectrometry (LC-MS/MS) enable either the targeted identification and quantification of BTXs for which chemical standards are available [18,31,32,33,34], or the non-targeted detection of potential new BTX analogues [35]. (2) Biochemical methods, such as specific binding tests (receptor binding assay (RBA) or immunological tests (radioimmunoassay (RIA), ELISA)), enable the overall quantification of BTXs [33,36,37,38]. (3) Biological methods (in vivo and in vitro), in particular the mouse bioassay [17], fish bioassay and neuroblastoma cell-based assay (Neuro-2a) [39,40], are able to determine the overall biological activity of BTXs. Several of these methods have been validated through intra- and inter-laboratory studies. However, none have been validated through inter-laboratory studies in accordance with the guidelines of standards such as ISO 5725. Therefore, to date, there are no standardized method for the detection of BTXs.

To prevent health risks associated with the consumption of shellfish contaminated with BTXs in France, a working group was set up by the French Agency for Food, Environmental and Occupational Health & Safety (Anses). The aims of this work were first, to examine toxicological data in order to identify a lowest observed adverse effect level (LOAEL) possibly appropriate to calculate an acute oral reference dose (ARfD); second, to propose a guidance level in shellfish to protect human consumers; third, to identify investigations required if the guidance level was exceeded; and finally to provide recommendations for the monitoring of BTXs in the marine environment.

## 2. Results

### 2.1. Detailed Cases of NSP Available in the Literature

There are few cases reported in the literature of human intoxications related to the ingestion of BTX-contaminated shellfish. A few hundred cases were described between 1962 and 2017 in international peer-reviewed journals, of which 48 cases (occurring over a few weeks in North Carolina) are from a single publication [41]. In New Zealand, 180 cases were reported between 1992 and 1993 in association with the consumption of mussels, clams, and oysters [5,42,43,44,45,46]. Between 1997 and 2010, poisoning cases were scarce in the United States, with only 21 reported cases of NSP, 20 of which (one was unknown) were related to recreational fishing during a red tide event [47]. 

Nineteen cases of NSP were reported following the ingestion of Florida oysters or clams in 1962 and 1963 [48]. The signs presented by a group of four cases are well documented. After ingestion of two-three raw oysters, no signs were first reported, but after ingestion of a cold drink, oral dysesthesia was triggered in one person. Later in the day, these four cases each consumed a total of 15–20 cooked oysters and all experienced oral, distal and/or generalized paresthesia, as well as a feeling of drunkenness and cold, one to three hours after ingestion. Some also presented diarrhea with rectal pain, abnormal perception of drinking temperature, bradycardia and mydriasis. A fifth case, who reported a consumption of 4 or 5 dozen raw oysters, exhibited three hours after ingestion oral and distal paresthesia, dizziness, nausea, ataxia, bradycardia, and diarrhea. The other cases (*n* = 14) showed symptoms described as similar. All clinical signs resolved within 24–48 h. In 14 cases, the number of oysters ingested and their toxicity, quantified by the mouse bioassay, were reported. It is not specified if the samples analyzed are all meal remnants, or not, but it is mentioned in the paper that “Since some of the oysters causing the human illnesses were still available, samples were sent […] for analysis”. The shellfish analyzed had a BTX level of 135, 100 and >65 MU/100 g of flesh.

In 1973 and 1974, a red tide event in Florida led to three series of NSP outbreaks, for 11 intoxicated people [49]. The first episode occurred in November 1973, with the consumption of surf clams (*Spisula solidissima raveneli*) near Sarasota (Florida) by two children aged 10 and 12 years old. Shortly after the meal, they developed abdominal pain, limb paresthesia and headaches. The state of health of the youngest child rapidly deteriorated, with seizures progressing to coma, and finally respiratory arrest requiring intensive care including intubation associated with mechanical ventilation. During the same month, five adults ate quahog (American clam *Mercenaria campechiensis*) collected from the same area and three of them exhibited mild typical symptoms of NSP, requiring hospitalization for one of them. Finally, a third episode of collective intoxication occurred, again in Florida, in four adults. The symptoms were mild in three of them, and moderate but questionable in the fourth. After all three episodes, clams were either obtained from the lots used in meal preparation or harvested at the original site of harvest and analyzed by the mouse bioassay. Shellfish analyses showed contamination between 75 and 118 MU/100 g. Bodyweight, number of shellfish eaten, and toxicity of the shellfish were available for each of the 11 cases. The authors [49] estimated a LOAEL of 0.3–0.4 MU per kg of body weight (MU/kg bw).

In 1987, in North Carolina, 48 out of 85 oyster consumers were intoxicated by BTXs [41]. The most common symptoms were paresthesia (81%), vertigo (60%), malaise (50%), abdominal pain (48%), nausea (44%), diarrhea (33%), weakness (31%) and ataxia (27%). Surprisingly, 17% of patients reported a reversal of temperature perception, a symptom not commonly reported in other intoxication events that happened in Florida, and which are more generally associated with ciguatera-type poisoning (reviewed in [50]). The mean time to onset of symptoms was three hours (interval between 15 min and 18 h), and the intensity of symptoms was associated with the number of shellfish ingested. Almost all the patients had multiple symptoms, and most of them had more than one neurological symptom. Only one patient was admitted to the hospital for exhibiting severe neurological signs: bilateral carpopedal tremor, myalgia, total body paresthesia, ataxia, and vertigo. There were no cases of respiratory distress. The mean duration of symptoms was 17 h (range 1–72 h). This case series represents the best clinical and epidemiological description of oral BTX poisoning reported in the literature. Samples of oysters eaten by four affected persons from two meals were analyzed (35 and 60 MU, respectively). Samples of oysters harvested from the same general areas as those that were eaten by all the other cases also tested positive (mean, 62 MU; range, 48–170 MU). Two out of 15 people who consumed less than 12 oysters exhibited symptoms (i.e., 13% attack rate in this consumer group). For a consumption of 12 or more oysters, the attack rate was 65% (45/69 people). Using 12 oysters in the calculation (threshold causing symptoms in a small proportion of people), and assuming a weight of 10 g per oyster, Gessner (2000) [51] provided an estimate of the toxic dose leading to mild symptoms ranging between 42 and 72 MU/person. This value is used in some reviews [2,52,53] but is very uncertain (see below).

Another serious event occurred when a family of three was poisoned in June 1996 after the consumption of gastropods and molluscs (whelks and clams) collected in Sarasota Bay (Florida) [54]. It was the first documented NSP event associated with the consumption of whelks. One of the parents experienced paresthesia in the face and extremities of the limbs, as well as vomiting. The two young children (aged 2 and 3 years) were hospitalized with severe neurological signs and symptoms (seizures, loss of consciousness), dyspnoea and tachycardia. Vomiting and abdominal muscles pain were also present. The two children were admitted into the intensive care unit; recovery occurred within days, but no long-term follow-up was performed. BTXs were identified in the urine of children by the RIA method (42 ± 2 ng/mL BTX for one child, and 117 ± 30 ng/mL for the second child). BTXs have also been detected by RIA in extracts of ingested molluscs and gastropods [54,55]. The father reported eating several whelks, and children ingested unknown amounts, but probably less.

In July 2005, in Florida, four people (two adults aged 31 and 34 years, two children aged 6 and 9 years) were hospitalized after consuming a meal of oysters harvested out of an area that was closed due to elevated *K. brevis* cell numbers [15,53]. The youngest child had episodes of seizures. His clinical condition required intubation associated with mechanical ventilation in an intensive care unit. Other symptoms reported were involuntary muscle spasms and cramps (all cases), abdominal pain, and paresthesia of the face and extremities of the limbs (reported for three of the four cases) and vomiting and headache (for two out of the four cases). Symptoms appeared quickly, and children were more severely affected than adults. No data were available to estimate ingested doses [53].

In 2006, in Southwest Florida, during a prolonged bloom of *K. brevis,* 20 NSP cases have been reported to the local health authorities. Cases appeared sporadically from March to December 2006, mostly in July, in relation with the consumption of clams harvested in recreational areas not open to legal shellfish harvesting, except one case related to consumption of conch (a gastropod) [15,53,55]. Symptoms from this episode appeared to be more severe than that reported in the 1997 episode in North Carolina. Disease registry data of Florida (the Florida Poison Information System) and telephone questionnaires were used to obtain clinical and demographic data. All cases of Florida poisoning reported 5 to 17 different symptoms, notably neurological. The most common reported symptoms (*n* = 20) were paresthesia in the lips and mouth (90%), paresthesia in the extremities (90%), nausea (80%), muscle weakness (80%), vomiting (65%), ataxia (65%), slurred speech (55%), dizziness (50%), and respiratory discomfort (35%). Diarrhea, fatigue, pain, muscle contraction, headache, cramping, partial paralysis, and severe neurological effects were reported to a lesser extent (20% to 30% of cases). Chest pain, blurred vision, sweating, respiratory distress, tachycardia, and fever were not often reported (in 10% of cases or less). Seventeen out of the 20 people sought medical treatment at local emergency departments of which seven (41%) were admitted to the hospital. One individual with underlying medical conditions was placed on ventilatory support, and fully recovered three days after. Another person was placed in the intensive care unit for a short period with severe neurological symptoms. In addition, some people reported spasms, muscle fasciculation and psychotic-like outbursts (unspecified, *n* = 6). Only one case in this Florida series reported an inversion of temperature perception. The differences in reported symptoms between Florida and North Carolina episodes may be due, in part, to variations in the methods used to document symptoms, and the time lag between the illness and the medical visit for some of the cases reported in Florida. The variations in composition of the BTX mixture between these two red tide events may also be responsible for the differences in symptoms, although this hypothesis cannot be retrospectively tested. Leftover meals (clams) were available for testing from a few of these 2006 Florida cases. The total BTX was 42.9 mg/kg and 24 mg/kg shellfish meat (determined by ELISA) in a cluster, well above the regulatory level of 800 μg/kg. Analysis of harvested clams from the same area as the implicated clams showed that they were also contaminated (23.6 mg/kg by ELISA).

Finally, in March 2017, two people were admitted to the emergency room in Florida following consumption of gastropods (“horse conch” *Triplofusus giganteus*) taken from an area affected by a prolonged *K. brevis* bloom from September 2016 to February 2017 [7]. In March 2017, *K. brevis* was not present, or was present at low levels (<10,000 cells/L), but the area had remained closed for the harvesting of bivalves. Gastropods have been eaten cooked (boiled in water 45 min). Symptoms appeared between 4 and 6 h after ingestion and lasted 24 to 34 h. Patients exhibited generalized weakness, unusual fatigue, body numbness, dizziness, reversal of the perception of hot/cold temperature, paresthesia, nausea, and ataxia. There were no leftover meals for analyzing BTX concentrations. Bivalve samples and gastropods were collected from the same site one week after the NSP cases. The concentrations measured by ELISA ranged from 1.1 to 198 mg BTX-3 eq./kg of tissue (flesh or viscera). Using LC-MS/MS, the metabolites BTX-B1 (Taurine BTX-B), BTX-B2 (Cysteine-BTX-B sulfoxide), S-deoxy-BTX-B2 (Cysteine-BTX-B), BTX-B5, BTX-2 and BTX-3 have been measured and quantified at lower concentrations than by ELISA (maximum at 58 mg/kg for the sum of metabolites in gastropod viscera). The urine of the patients was analyzed and the presence of BTX was confirmed by ELISA and LC-MS/MS.

Based on these detailed cases, NSP is caused by the consumption of BTX-contaminated molluscs, mainly bivalves (oysters, clams) [42,48,49,54], but also whelks [7,15,53,54,55]. The symptoms and their frequency are reported in Table 1. They generally occur one to 24 h after exposure and can last up to three days. NSP is primarily characterized by the occurrence of neurological disturbances, which may be associated with gastrointestinal and/or cardiovascular symptoms. The digestive signs include abdominal pain, nausea, vomiting and diarrhea. The neurological signs mainly consist of paresthesia (lips and extremities), dizziness, asthenia, muscle disorders, speech impairment, loss of coordination and coma in the most serious cases. Reversal of temperature sensation, mydriasis, and cardiovascular disorders (including bradycardia and arterial hypotension) have also been reported. No deaths have been reported.

Symptoms associated with the lowest doses of BTXs ingested are paresthesia in the peri-oral region and extremities, and dysesthesia in contact to cold drink. Two studies [48,49] provided sufficient data to estimate the ingested doses associated with these symptoms, i.e., LOAELs as a starting point for the hazard characterization of BTXs after acute oral exposure.

### 2.2. Hazard Characterisation

Data on acute toxicity in animals are very limited and do not enable the identification of a no observed adverse effect level (NOAEL) or a lowest observed adverse effect level (LOAEL). The only study by oral administration aimed to determine median lethal doses (LD_50_) for BTX-2 and BTX-3 in female mice [56]. Oral LD_50_ was 6600 µg/kg bw (IC95: 2900–14,800 µg/kg bw) for BTX-2 and 520 µg/kg bw (IC95: 370–730 µg/kg bw) for BTX-3. By oral administration (gavage), BTX-3 was 10 times more toxic than BTX-2. But by intraperitoneal injection, the two toxins were almost equipotent (i.p. LD_50_ of 200 (IC95: 150–270) and 170 (IC95: 140–210) µg/kg bw, respectively for BTX-2 and BTX-3). Due to this difference, Toxic Equivalency Factor (TEF)–required to estimate toxicity in shellfish using chemical methods–should be based on oral toxicity data.

However, a test of lethality cannot be used as the starting point for an acute reference dose (ARfD) because it would not be protective enough.

Data on acute toxicity in humans were too limited to enable the establishment of an ARfD. Nevertheless, based on data associated with human NSP cases after consumption of BTX-contaminated shellfish, we identified lowest levels (ingested quantities of BTXs) associated with symptoms (“acute LOAELs”), and minimum concentrations in shellfish associated with symptoms, which are presented in Table 2.

A mouse unit (MU) is the amount of raw extract that kills 50% of mice (20 g) within 930 min (15.5 h) [17]. One MU = 3.4 µg BTX-3 or 4 µg BTX-2, according to Baden and Mende (1982) [56].

Based on the data reported by Morris (1991) [41], Gessner (2000) [51] estimated the level causing minor symptoms at 42 to 72 MU/person. This value is often used in reviews [2,52,53], however we consider it as highly uncertain. Indeed, this value is based on a threshold consumption level causing symptoms in a low proportion of individuals (low consumer group) and on the contamination levels measured in leftovers from only 2 meals (35 and 60 MU/100 g). These meals concerned only 4 of the 48 quantified cases. The article by Morris (1991) [41] did not state the consumption level actually consumed by these 4 cases, or the contamination level of the oysters ingested by the low consumer group.

We found the studies by McFarren et al. (1965) [48] and Hemmert (1975) [49] particularly relevant because they contain detailed individual information on symptoms, portion sizes, body weights (for one study) and BTX quantification in leftovers. The data allowed identifying two “acute LOAELs”: 27–40.5 MU/person recalculated by us based on McFarren et al. (1965) (we assumed a 10 g oyster flesh weight rather than 20 g assumed by McFarren et al. (1965) and 0.3–0.4 MU/kg bw reported by Hemmert (1975) (Table 2).

We selected BTX-3 (and not BTX-2) as the reference BTXs in shellfish, for the following reasons: (i) BTX-3 is the reference BTX for the ELISA test; (ii) BTX-3 has a lower oral LD_50_ value than BTX-2, and (iii) BTX-3 was reported at higher level than BTX-2 in shell-fish [4,5,6,7,8,9]. The maximum level of 20 MU/100 g or 800 µg BTX-2/kg corresponds to 680 BTX-3 eq./kg shellfish flesh.

In BTX-3 equivalents (1 MU = 3.4 µg BTX-3 [56]), the “acute LOAELs” would be 92–138 µg BTX-3 eq./person based on McFarren et al. (1965), and 1.02–1.36 µg BTX-3 eq./kg bw based on Hemmert (1975).

### 2.3. Recommended Guidance Level in Shellfish

To assess the degree of protection provided by the Codex Alimentarius [16] maximum level of 20 MU/100 g (800 µg BTX-2/kg shellfish flesh), we calculated the exposure levels associated with the consumption of shellfish contaminated at this level using several consumption assumptions, and compared them to the two selected “acute LOAELs” (Table 3). The consumption assumption included the consumption level of 400 g of shellfish flesh per person set by EFSA to protect the largest shellfish consumers [58], as well as several high consumption levels based on a consumption survey on seafood products in France (CONSOMER) conducted in 2016–2017, as part of a research agreement between ANSES and CREDOC (2015-CRD-25). The aim of the survey was to assess seafood consumption by an adult population (over 18 years of age) living in coastal areas and with access to local markets. The CONSOMER database includes answers from 2481 adults.

Based on the “acute LOAEL” of 0.3–0.4 MU/kg bw reported by Hemmert (1975) [49], with an assumed body weight of 70 kg and a protective default consumption of 400 g of shellfish flesh [58], we calculated a level of 52.5–70 MU/kg shellfish flesh. According to Baden and Mende (1982) [56], 1 MU = 3.4 µg BTX-3, which would correspond to a concentration of 179–238 µg BTX 3 eq./kg shellfish flesh. This is three to four times lower than the Codex maximum level (20 MU/100 g or 800 µg BTX-2/kg, or 680 BTX-3 eq./kg shellfish flesh).

Based on the “acute LOAEL” of 27–40.5 MU/person calculated here from the study by McFarren et al. (1965) [48], and assuming a protective default consumption of 400 g of shellfish flesh [58], we calculated a level of 67.5–101.25 MU/kg shellfish flesh, i.e., a concentration of 230–344 µg BTX-3 eq./kg shellfish flesh. This is two to three times lower than the Codex maximum level.

In conclusion, the Codex maximum level of 20 MU/100 g shellfish flesh does not appear protective enough. We therefore recommend a guidance level of 180 µg BTX-3 eq./kg shellfish flesh, based on the lowest value of the range of “acute LOAEL” [49]. It was not deemed necessary to apply an additional safety factor due to the protective assumptions on which the calculations were based (default consumption of 400 g of shellfish flesh and a 70 kg bw). This guidance level applies to the sum of all tested BTX metabolites by LC-MS/MS analysis for which standards are available, or to the results of ELISA test expressed in BTX-3 eq.

## 3. Discussion

The first detection of BTXs (BTX-2 and/or BTX-3) in France was in November 2018, with a maximum level, observed in November 2020, of 57 µg/kg total mussel flesh for the sum of BTX-2 and BTX-3 by LC-MS/MS [18]. Detection of BTXs in France has so far only been observed in mussels from the Diana lagoon Eastern coast in Corsica (Figure 1). No BTXs were detected in oysters sampled at the same location site. In addition, a retroactive analysis of preserved mussels demonstrated the presence of BTX-3 in mussels from the same site sampled in November 2015. The detection of BTXs could be related to the presence in seawater samples at the same period in the lagoon of four *Karenia* species (*K. mikimotoi*, *K. papilionacea*, *K. longicanalis,* and an undescribed species *Karenia* sp. 1), and two raphidophytes (*Fibrocapsa japonica* and *Heterosigma akashiwo*), which are all potential BTX producers. No *Karenia brevis* cells have been observed in France so far. It remains therefore essential to conduct further investigations to identify the BTX-producing algal species in Corsica Island.

In the United States, monitoring systems were set-up, particularly in Florida, Texas, Delaware and Alabama. The risks associated with blooms of the *Karenia* genus are monitored and controlled via the regular monitoring of *K. brevis* in water. BTXs are monitored in shellfish and air, or in response to an episode of fish mass mortality, or when respiratory symptoms have been reported in humans. Citizen science networks are also involved in this monitoring. In the United States, Mexico, Australia and New Zealand, the health authorities have established thresholds: (a) for the number of microalgae cells in water that can lead to preventive management measures; (b) for BTXs in shellfish flesh requiring the closure of production areas [11,12,13,14].

In France, BTXs are currently not regulated. *Karenia* genus are included in the French Observation and Monitoring program for Phytoplankton and Hydrology in coastal waters (REPHY), but BTXs are not systematically analyzed in shellfish, as part of the French Monitoring program for Phycotoxins in marine organisms (REPHYTOX). BTXs are among the emerging toxins monitored by the EMERGTOX network, only once a month on a limited number of shellfish production areas.

Data on acute oral toxicity in animals are very limited and do not allow the identification of a NOAEL or a LOAEL. In humans, the data were too limited to enable the establishment of an ARfD. Nevertheless, based on data associated with human NSP cases after ingestion of BTX-contaminated shellfish, we identified two lowest level ranges (ingested quantities of BTXs) associated with symptoms (“acute LOAELs”). Despite the uncertainties associated with their estimation, both ranges were of the same order of magnitude, and were therefore considered relevant to derive a guidance level for the presence of BTXs in shellfish.

Based on the “acute LOAEL” of 0.3–0.4 MU/kg bw reported by Hemmert (1975) [49], we calculated a concentration of 179–238 µg BTX-3 eq./kg shellfish flesh which is approximately three to four times lower than the Codex maximum level [16] equivalent to 680 BTX-3/kg shellfish flesh. Based on the “acute LOAEL” of 27–40.5 MU/person recalculated by us from the study by McFarren et al. (1965) [48], we calculated a concentration of 230–344 µg BTX-3 eq./kg shellfish flesh which is approximately two to three-fold lower than the Codex maximum level. In conclusion, we considered that the maximum level of 20 MU/100 g shellfish flesh (equivalent to 680 BTX-3 eq./kg shellfish flesh) is not protective enough. Based on the lowest value derived from our calculations, we recommend a guidance level of 180 µg BTX-3 eq./kg shellfish flesh. This guidance level applies to the sum of all tested BTX metabolites by LC-MS/MS analysis for which standards are available, or to the results of ELISA test expressed in BTX-3 eq.

In the event that this guidance level is exceeded, in mussels or oysters in Corsica (species regularly monitored by EMERGTOX at the Diana site, the only area of metropolitan France affected by BTXs to date), we recommend: (i) to provide information to healthcare professionals to improve the detection and declaration of potential NSP cases; (ii) to test for the presence of BTXs in the other shellfish species potentially produced in the affected area; and (iii) to test for the presence of BTXs in surrounding shellfish production sites.

In addition, we identified the following data gap that should be addressed to improve the risk assessment related to BTX oral exposure.

Oral toxicity data from animal studies to be used as the starting point for an ARfD are currently lacking. It is therefore recommended to assess the effects of BTXs by an acute oral toxicity study in rodents conducted according to the OECD Guideline 424 for neurotoxicity testing (single administration, 14-day observation period). This study should describe all visible clinical symptoms (in particular behavioral changes such as hyper-reactivity) and evaluate biological parameters (respiratory and cardiovascular parameters, monitoring of internal temperature, gastrointestinal effects) as thoroughly as possible, to determine the dose-effect relationship. Such a study would help defining a critical effect of acute poisoning in rodents and identify a no observed effect level as the starting point for calculating a health-based guidance value. This recommendation should apply firstly to BTX-3 and then to other major BTXs present in shellfish.

Given the similarity between BTXs and ciguatoxins, the neurotoxins responsible for ciguatera, whose effects can last several months or even years following acute exposure [50], we consider that long-term effects following acute oral exposure to BTXs cannot be ruled out.

To the best of our knowledge, no repeated-dose oral toxicity studies have been conducted, meaning that it is not possible to propose a chronic health-based guidance value. Information is also lacking regarding the potential reproductive and developmental toxicity of BTXs.

Metabolites of BTXs in shellfish for which there are no data on toxic potential should be studied, as first step, in vitro (RBA and/or Neuro-2a). In this aim, sufficient quantities of these compounds should be isolated and purified, and their structure identified.

Studies should be undertaken on the relative toxicity of major BTXs in shellfish compared with BTX-3 to determine toxic equivalency factors (TEFs).

The effects of mixtures of BTXs should also be investigated.

There is a need for a reliable acute reference dose, an internationally harmonized regulatory limit, standardized analytical methods, certified reference materials, and robust toxicity equivalence factors.

## 4. Materials and Methods

### 4.1. Analysis of the Literature

The work was based on a scoping review of the literature. Two databases (Scopus and PubMed) were queried on 14 May 2020 with the term “Brevetoxin*” in the field “Title–Abstract–Key words” for Scopus and “All fields” for Pubmed, without date restriction. This search resulted in 868 references (once duplicates have been removed), exported to EndNote software. The references were screened for inclusion/exclusion criteria based on title and abstract (sometimes full text, when needed). The inclusion criteria were (i) studies on BTXs, (ii) studies on absorption/distribution/metabolism/excretion of BTXs, (iii) toxicity of BTXs in humans, (iv) toxicity of BTXs in vivo or in vitro, and (v) exposure to BTXs. The exclusion criteria were (i) studies on toxins other than BTXs, (ii) studies on chemical synthesis of BTXs, (iii) language other than English and French, and (iv) full-text not available. An update of the literature search was conducted in October 2020, identifying four additional publications, as well as one article posted in December 2020, which have been added to the body of references. After the first screening step, the remaining 515 references were reviewed by all the members of the working group to identify relevant studies. Each reference has been assessed by at least two reviewers. The working group met 7 times between June 2020 and January 2021.

Sixty-four references were identified as relating to in vivo toxicity. However, data on oral acute toxicity in animals were very limited and did not allow the identification of a NOAEL or a LOAEL. The only studies by oral administration were indeed aimed at determining the LD_50_ for BTX-2 and BTX-3 [56]. This measure was considered as not protective enough to be used as the starting point for an ARfD.

### 4.2. Guidance Level Calculation

Twenty-eight references were identified as relating to acute oral toxicity in humans (NSP cases), of which six were informative on reported symptoms (sometimes at individual level), contamination of shellfish and for some cases on estimated doses associated with symptoms. These six studies were analyzed to identify lowest levels (ingested quantities of BTXs) associated with symptoms (“acute LOAELs”). These results were expressed in mouse unit (MU) per person, where a MU is the amount of raw extract that kills 50% of mice (20 g) within 930 min (15.5 h) in the mouse bioassay used to quantify extract toxicity [17]. One MU = 3.4 µg BTX-3 or 4 µg BTX-2 [56]. Based on the selected “acute LOAELs”, we calculated the maximum level of BTXs in shellfish to protect shellfish consumers using the protective default portion size of 400 g of shellfish flesh per person set by EFSA [58]. That is to say that LOAELs expressed in MU/person [48] were multiplied by 2.5 (=1000 g/400 g) to be converted in MU/kg shellfish meat. For LOAELs expressed in MU/kg bw [49], we used a default body weight of 70 kg to convert the LOAELs in MU/person and then applied a 2.5 factor, as previously. To convert MU in equivalents of BTXs, we selected BTX-3 (and not BTX-2) as the reference BTX in shellfish, for the following reasons: (i) BTX-3 is the reference BTX for the commercialized ELISA test kit; (ii) BTX-3 has a lower oral LD_50_ value than BTX-2, and (iii) BTX-3 was reported at higher level than BTX-2 in shellfish [4,5,6,7,8,9]. The maximum level of 20 MU/100 g or 800 µg BTX-2/kg corresponds to 680 BTX-3 eq./kg shellfish flesh.

## Figures and Tables

**Table 1:** Clinical features reported in NSP outbreaks.

Signs and Symptoms	Outbreak Location, Date, Number of Cases (n) and Reference				
	Florida (1962) *n* = 5 [48]	Florida (1973–74) *n* = 11 [49]	North Carolina (1987) *n* = 48 [41]	Florida (1996) *n* = 3 [54]	Florida (2006) *n* = 20 [53]
Abdominal pain		45%	48%	x	
Pain (undefined location)					20–30%
Nausea	20%		44%	x	80%
Diarrhea	60%		33%		20–30%
Vomiting			10%	x	65%
Paresthesia ^1^	100%	55%	81%	x	90%
Feeling cold	40%				
Dysesthesia ^2^	40%		17%		5%
Myalgia ^3^			13%		
Vertigo ^4^	80%	36%	60%		50%
Ataxia ^5^	40%		27%		65%
Tremor			6%		
Muscle weakness					80%
Cramping					20–30%
Muscle contractions		9%			20–30%
Rectal pain	20%				
Asthenia			31%	x	20–30%
Malaise			50%		
Chills			21%		
Headache		9%	15%		20–30%
Mydriasis ^6^	80%				
Blurred vision					≤ 10%
Bradycardia	40%				
Slurred speech					55%
Partial paralysis		9%			20–30%
Respiratory discomfort					35%
Chest pain					≤10%
Sweating					≤10%
Fever					≤10%
Respiratory distress		9%		x	≤10%
Tachycardia				x	≤10%
Loss of consciousness				x	
Convulsions		9%		x	
Seizures				x	
Coma		9%			
Sever neurological symptoms					5%
Decerebrate posturing ^7^		9%			

Empty cells: no information available; x: details not available. ^1^ Paresthesia: abnormal sensation, whether spontaneous or evoked, that is not unpleasant (according to the International Association for the Study of Pain, IASP) (e.g., numbness or painless tingling or stinging sensation). ^2^ Dysesthesia: abnormal sensation, whether spontaneous or evoked, that is unpleasant or painful (according to the IASP) (e.g., burning, pricking sensation, painful tingling). ^3^ Myalgia: muscle pain. ^4^ Vertigo: loss of balance, dizziness. ^5^ Ataxia: disorders that affect co-ordination, balance, and speech. ^6^ Mydriasis: dilatation of the pupil. ^7^ Decerebrate posture is an abnormal body posture that involves the arms and legs being held straight out, the toes being pointed downward, and the head and neck being arched backward. The muscles are tightened and held rigidly. This type of posturing usually means there has been severe brain damage.

**Table 2:** Lowest levels of BTXs associated with symptoms (“acute LOAELs”) and minimum concentrations in shellfish associated with symptoms (see Section 2.1 for details on the symptoms).

Studies	Lowest Levels with Symptoms (“Acute LOAELs”)	Corresponding Minimum Concentrations in Shellfish Flesh Associated with Symptoms
McFarren et al. (1965) [48]	405–540 MU/person for moderate symptoms 54–81 MU/person induced minor symptoms (paresthesia) in one case (we revised this level to **27**–40.5 MU/person *) 91 MU/person for moderate symptoms	135 MU/100 g 135 MU/100 >65 MU/100 g
Hemmert, 1975 [49]	**0.3**–**0.4 MU/kg bw**	75–118 MU/100 g
Morris, 1991 [41]	n.a	35 and 60 MU/100 g
Watkins, 2008;Terzagian, 2006 [53,55]	n.a	24 and 42.9 mg BTX-3 eq./kg (ELISA)
For comparison, the maximum level used by the Codex Alimentarius, US FDA, Australia/New Zealand and Mexico [11,12,13,14,16]		20 MU/100 g 800 µg BTX-2/kg

MU: mouse unit; n.a: not applicable. * McFarren et al. (1965) used a flesh weight of 20 g per oyster. Based on [57] we considered that a flesh weight of 10 g/oyster was more appropriate than 20 g/oyster (meat yield for *Crassostrea virginica* ranging from 0.09 to 0.22). The lowest level associated with symptoms would be 27–40.5 MU/person (two or three oysters with 10 g of flesh, contaminated at a level of 135 MU/100 g flesh).

**Table 3:** Assessment of the protective nature of the maximum level (ML) of 20 MU/100 g (800 µg BTX-2/kg shellfish flesh or 680 µg BTX-3 eq./kg shellfish flesh) comparing the calculated lowest dose with symptoms (“acute LOAEL”) against the estimated exposure of shellfish contaminated at the maximum level with different portion sizes. **Bold** indicates values above the estimated “acute LOAEL”.

		Exposure (MU/Person)	Exposure (μg BTX-3 eq./Person)
**“acute LOAEL”**	Hemmert (1975) ^a^ [49] Mc Farren et al. (1965) ^a^ [48]	21–28 ^b^ 27–40.5 ^c^	71.4–95.2 91.8–137.7
Shellfish at CODEX maximum permitted level (20 MU/100 g)	French consumption of clams ^d^		
	P95 = 50 g P97.5 = 60 g	10 12	34 68
	French consumption of oysters ^d^		
	P95 = 182.4 g P97.5 = 255 g	**36.5** **51**	**124.1** **173.4**
	French consumption of mussels ^d^		
	P95 = 200 g P97.5 = 300 g	**40** **60**	**136** **204**
	Large portion size of 400 g [58]	**80**	**272**

^a^. The estimations are based on calculation assumptions made by both the original authors and us, each associated with a margin of error that is difficult to estimate. The values in Table 2 should therefore be considered with a moderate level of uncertainty for the study by Hemmert (1975) and with a high level of uncertainty for that by McFarren et al. (1965). ^b^. To convert the LOAEL from Hemmert (1975) from MU kg/bw to MU/person, we used a default bodyweight of 70 kg. ^c^. Adjusted by us based on 10 g flesh weight for oyster rather than 20 g by Mc Farren et al. (1965). ^d^. Shellfish portion sizes from the CONSOMER 2016–2017 consumption survey (Anses data).
